# The association between care left undone and temporary Nursing staff ratios in acute settings: a cross- sectional survey of registered nurses

**DOI:** 10.1186/s12913-020-05493-y

**Published:** 2020-07-10

**Authors:** Michaela Senek, Steve Robertson, Tony Ryan, Rachel King, Emily Wood, Angela Tod

**Affiliations:** Division of Nursing & Midwifery, Department of Health Sciences, University of Sheffield, Sheffield, UK

**Keywords:** Nurse staffing, Temporary staff, Care left undone, Acute settings

## Abstract

**Background:**

The shortage of health workers is a global phenomenon. To meet increasing patient demands on UK health services, providers are increasingly relying on temporary staff to fill permanent posts. This study examines the occurrence of ‘care left undone’, understaffing and temporary staffing across acute sector settings.

**Methods:**

“Secondary data analysis from an RCN administered online survey covering nurses from hospitals and trusts across all four UK countries. Staffing and ‘care left undone’ measures were derived from the responses of 8841 registered nurses across the UK. A locally smoothed scatterplot smoothing regression analysis (Loess) was used to model the relationship between any ‘care left undone’ events and full complement, modest and severely understaffed shifts, and proportions of temporary staff.

**Results:**

Occurrence of ‘care left undone’ was highest in Emergency Departments (48.4%) and lowest in Theatre settings (21%). The odds of ‘care left undone’ increase with increasing proportion of temporary staff. This trend is the same in all understaffing categories. On shifts with a full quota of nursing staff, an increase in the proportion of temporary staff from 0 to 10% increases the odds of care left undone by 6% (OR = 1.06, 95% CI, 1.04–1.09). Within the full quota staffing category, the difference becomes statistically significant (p < 0.05) on shifts with a proportion of temporary nursing staff of 40% or more. On shifts with a full quota of nursing staff the odds of a ‘care left undone’ event is 10% more with the proportion of temporary nursing staff at 50%, compared to shifts with modest understaffing of 25% or less with no temporary nursing staff (OR = 1.1, 95%CI, 0.96–1.25).

**Conclusion:**

The odds of a ‘care left undone’ event are similar for fully staffed shifts with a high temporary nursing staff ratio compared to severely understaffed shifts with no temporary nursing staff. Increasing the proportion of temporary nurse staff is associated with higher rates of self-reported care left undone by nursing staff. This has significant implications for nurse managers and policy makers.

## Background

The global shortage of professional health care staff can have adverse effects on the delivery of health care [1]. The World Health Organisation (WHO) estimates that, by 2030, there will be a worldwide net shortage of 15 million doctors, nurses and other health workers [2]. The WHO has identified this as one of the most pressing global health issues of our time. Where modelling of nurse workforce supply has occurred, four out of five predict shortages in the medium term (UK, Ireland, Canada and Australia) [3]. The US predicts nurse over supply [4].

The focus of this research is the UK health service workforce, where staffing shortages within the National Health Service (NHS) are prevalent. A recent press release by the Health Foundation, King’s Fund and Nuffield Trust predicts that the current gap of 40,000 nursing vacancies could worsen significantly, with nurse shortages doubling by 2023/24 [5].

To address rising patient demand and nursing rotation gaps, health care providers across the UK are increasingly reliant on temporary staff to fill permanent posts. As a result, the number of care hours provided by non-permanent registered nurses more than doubled from 930,000 in 2012 to 1,917,000 in 2015 [6]. The King’s Fund has classed it a “continuing problem with staffing levels, which Trusts [NHS hospital providers] are solving by using temporary nursing staff in the absence of sufficient permanent workers,” [6]. The concept of missed nursing care (or ‘care left undone’) was developed by Kalisch & Williams [7]. They identify missed care as ‘an error of omission and is defined as any aspect of required patient care that is omitted (either in part or completely) or significantly delayed’ (p. 291). The concept has been further developed by more recent work undertaken by the RN4CAST team, informed by the concept of ‘nursing things left undone’ [8]. RN4CAST carried out a European wide survey using a structure, process and outcome model as the theoretical underpinning for the work. Structure referred to ‘work environment’ and ‘staffing levels’ as important antecedents in the process of nursing care. The subsequent study identified the significant prevalence of ‘care left undone’ in acute hospital environments across all participating countries [9].

Whilst several studies have examined the relationship between understaffing and ‘care left undone’, only a few studies have explored the association between types of nursing staff (temporary or permanent) and quality of care. Primarily, it has been found that sub-optimal numbers of registered nurses result in ‘care left undone’ and adverse patient outcomes. Staffing data measured as hours per patient day (HPPD), was found to be a significant predictor of missed care and a concomitant predictor of patient outcomes, beta = 0.45, *p* = 0.02 [10]. Higher registered nurse staffing levels were associated with fewer occurrences of missed nursing care. Similarly, a literature review by Griffiths et al., (2018) demonstrated the link between missed care and patient mortality. The review found that an increase in 10% of missed care resulted in a 16% increase in the odds of a patient dying within 30 days of admission (Griffiths et al., 2018). Other than adequate staffing, poor communication and teamwork have been implicated in adverse events [11]. A cross-sectional MISSCARE survey [12] showed that 38% of missed care cases were due to poor communication whilst higher levels of teamwork were associated with less missed nursing care. In addition, a high staff turnover has been shown to make teamwork in healthcare particularly challenging [13].

Similarly, a US study that included a sample of 2216 nursing staff members on 50 acute patient care units, found that the level of nursing teamwork affects the extent and nature of missed nursing care. The findings show that poor quality of teamwork alone accounted for about 11% of missed nursing care [14].

Importantly, these very same issues of communication and teamwork difficulties have been shown to be present when temporary RN staff are present. There is also evidence that the risk from falls, medication errors and nurse back injury are associated with higher levels of temporary RN staffing [15]. In the UK, research that examined ward patient dependency, nursing activity, workload, and staffing in 605 general and specialist wards between 2004 and 2009 found that workloads and time out (sickness absence, etc.) in wards employing temporary staff were greater than in units with permanent staff only. It also found that working styles were different and recommended that ward managers monitor temporary staffing levels and the effect they have on nursing activity and quality [13]. This is therefore an important area of concern when looking at the relationship between staffing levels and missed care. It is also important to note that many countries have roles such as licensed practical nurses, nursing associates and assistant practitioners as well as health care assistants to support registered nurses. As Dall Ora et al. evidence, these staff, whether permanent or temporary, may also have an impact on the prevalence of missed care, though this level of detail was not considered for the current analysis.

The UK National Health Service (NHS) has two types of temporary staff: bank nurses (usually a hospital’s own employees who work additional hours, often in their own clinical area, when required) and agency nurses provided by commercial recruitment agencies. Agency nurses are usually less familiar with specific ward patients and procedures than permanent ward staff [13]. Our aim in this study is to consider various acute care settings in order to explore the interaction effect between permanent, agency staffing ratios and ‘care left undone’. Findings from this study may help inform decisions about nurse staffing levels in hospitals with high proportions of temporary agency staff.

## Methods

This is a cross-sectional study analysing the prevalence of self-reported ‘care left undone’ and staffing resources, including the possible interaction effect between the use of agency nurses and scenarios where the number of registered nurses varies. It seeks to examine the association between staffing levels, proportion of permanent and agency nursing staff and prevalence of ‘care left undone’ in adult acute settings. In the UK, adult acute care covers all aspects of medical and surgical hospital in-patient care for those over 18 years of age but does not usually include in-patient mental health care.

This is a secondary analysis of existing data. The source of data is an online survey of registered nurses (RNs) developed and administered by The Royal College of Nursing (RCN) in 2017 in all four countries in the UK. Data was collected between the 14th May and 30th May 2017. The survey was administered via email and social media and was open to both members and non-members of the RCN. A report from the survey, produced by the RCN and covering all questionnaire domains, is available on the RCN website [16].

### Study population

The RCN is the UK’s largest professional nursing body consisting of 450,000 members of registered and non-registered nursing and health care staff. The original survey data comprised of 29,345 RNs from all four countries of the UK. The focus of this research was on the adult acute setting and the final findings comprised responses relating to 13,218 staff who worked in this sector. The respondents identified predominantly as ‘staff nurse’ (71.8%, *N* = 9490), 22.4% (*N* = 2960) identified as sister/charge nurse, 3% (*N* = 397) as clinical nurse specialist and 2.9% (*N* = 396) as senior nurse. We included RNs working in Emergency Department (ED), Adult Acute, Critical Care, Older People’s ward and Theatre. The questionnaire did not ask the respondents to identify the specific hospital that they worked in for reasons of anonymity. As a result, we are unable to carry out our analysis at the level of hospital and NHS trusts.

We identified from the dataset, and then excluded staff working in public health, outpatient, neonatal, maternity, inpatient children & young people, inpatient learning disability, inpatient mental health environments. This is to enable us to focus our analysis on adult acute care environments only. We also excluded respondents who identified themselves primarily within managerial non/clinical roles such as managers, team leaders, chief nurses, matrons and Associate Directors of nursing. We did this because such roles are usually removed from the immediate clinical environment and respondents may not have held adequate information about ‘care left undone’.

### Data Sharing Agreement and Ethical Approval

A data sharing agreement was obtained between The University of Sheffield and the RCN. The RCN-administered survey was then shared with a research team within University of Sheffield. All data was anonymised prior to being shared. Ethical approval was obtained on 27/08/2019 from the University of Sheffield (Reference Number 026774) to conduct a secondary analysis of the RCN survey.

### Measured outcomes

The survey asked respondents to provide information about their most recent working shift. Respondents were requested to provide information about the *planned* vs *actual* number of RNs, and proportion of *permanent* vs *temporary* agency RN numbers. These data were used to determine the *staffing levels and RN agency ratio* outcomes.

The binary outcome of ‘care left undone’ was based on respondents’ rating of the quality of care provided on their last shift. Respondents rated the statement; *Due to the lack of time, I had to leave necessary care undone* on a five-point scale (*strongly agree, agree, neither agree nor disagree, disagree and strongly disagree). “Necessary care left undone”* was noted to have occurred where the response was ‘agree’ or ‘strongly agree’. In order to clarify whether there was or was not care left undone, this scale was made into a binary outcome. The middle option on the Likert Scale ‘neither agree nor disagree’ was treated as a ‘missing value’. Data was also available on two important structural variables: staffing as a ratio of RN present to RN planned, and RN agency staff as a proportion of the RN staff present within a reported shift.

### Data analysis

Analysis of the prevalence of care left undone data used descriptive statistics in SPSS Version 25. We explored the data to identify distribution of responses, trends and outliers. Data were first analysed with descriptive statistics using proportions for categorical variables. The Shapiro–Wilk test was used to detect the presence of non-normality. For data where normal distribution could not be assumed, descriptive statistics were expressed as Median (Mdn) and interquartile range (IQR). A Mann Whitney U test was used to compare staffing ratio and permanent staffing ratio for ‘care left undone’ and ‘no care left undone’ shifts. Correlation was assessed using Spearman’s rho test. A probability below 0.05 (*p* < 0.05) was considered to be a significant difference.

We were interested in the relationship between the occurrence of any ‘care left undone’ events during the shift (a binary outcome) and two predictor variables, namely “Understaffing” (1 minus the ratio of actual to planned number of registered nurses) and “Agency ratio” (ratio of agency to total actual registered nurses working the shift). The relationship between ‘care left undone’ and staffing ratios data was explored using a locally smoothed scatterplot smoothing (“Loess”) regression in order to reveal relationships between the predictor variables and the outcome. Understaffing categories were grouped into three categories namely; full complement, 0.01–0.24 and ≥ 25 by agency ratio 0–0.1, 0.2–0.3 etc. By full complement, we mean that the scheduled number of registered nurses corresponds to the actual number of nurses present. Loess smoothing was preferred over a simple linear logistic regression model in order to accommodate any non-linear patterns in the data. All data processing, exploration and modelling were carried out in R statistical tool version 3.6.0.

## Results

For the purpose of analysing the relationship between care left undone and permanent staff ratio, of the 13,218 shifts, 4084 (31%) had missing data for at least one of the three variables of interest (understaffing, care left undone, agency proportion). The majority of these (23%) had a missing value for ‘care left undone’ or ‘neither agreed or disagreed/chose not to say’. A further 226 shifts with understaffing less than zero, seven shifts with Agency ratio greater than one, and 60 with Agency ratio equal to one, were excluded from analysis due to concerns over data quality. Therefore, data from 8841 shifts were included in the analysis.

### Prevalence of care left undone by setting

An important area of interest was for us to identify those clinical areas experiencing the highest prevalence of ‘care left undone’. The proportion of our responses by setting were; 15.3% from ED, 53.9% Adult Acute ward, 14% Critical Care, 11.2% were Older People’s ward and 5.6% Theatre. Within these responses, the highest proportion of “care left undone” was within the ED setting (48.4%) and lowest proportion within Theatre setting (21%, *N* = 122) (See Table 1). Adult acute ward reported, ‘care left undone’ on 45.3% of shifts (*N* = 2530), Older People’s Ward 46% (*N* = 535), Critical Care; 27.7% (*N* = 401).

**Table 1:** Staffing Levels and Self-reported Care Left Undone by Setting

Setting	Care Left Undone			
	Care Left Undone N (%)	No Care Left Undone N (%)	Neither Agree or Disagree (not included in the final analysis)	Total cases(N)
**Emergency Department/ Urgent and Emergency Care**	**763 (48.4%)**	**590 (37.3%)**	**225 (14.3)**	**1578**
**Adult Acute Ward**	**2530 (45.3%)**	**2244 (40.3%)**	**805 (14.4%)**	**5579**
**Critical Care/ high dependency**	**401 (27.7%)**	**854 (58.9%)**	**194 (13.4%)**	**1449**
**Older people’s ward**	**535 (46%)**	**438 (37.8%)**	**188 (16.2%)**	**1161**
**Theatre**	**122 (21%)**	**364 (63%)**	**93 (16%)**	**579**

There was no significant difference in reported outcome ‘care left undone’ across the four countries. The rates of care left undone were; Scotland 51%, Wales 49.3%, England 49.2% and Northern Ireland 48.6%.

Having established that some clinical areas are vulnerable to ‘care left undone’, our next task was to focus on those resource related factors that may have contributed to this. Subsequently we ‘pooled’ data from all five clinical areas to ensure that the tests were adequately powered. Staffing ratio on shifts that reported ‘no care left undone’ was higher than on shifts that reported ‘care left undone’ Mdn =0.94 (0.87–0.88) vs Mdn = 0.81 (0.79–0.80), *p*<0.001 (Mann Whitney U test). There was a moderate, positive relationship between staffing ratio and care left undone rs (4942) =0.25, *p* <0.05.

Further, we explored the relationship between the permanent staff ratio and care left undone on full complement shifts. The proportion of permanent staff was higher on shifts that reported ‘no care left undone’ (Mdn = 0.94 (IQR 0.8–0.82) than on shifts that reported ‘care left undone’ Mdn = 0.82 (IQR 0.72–0.74), *p* < 0.05. Spearman’s rho correlation was carried out to assess the relationship between proportion of permanent staff and care left undone. There was significant evidence of a moderate correlation between proportion of permanent staff and care left undone on full complement shifts rs (8816) = 0.5, *p* < 0.01).

Furthermore, we were interested in the prevalence of staffing scenarios within our sample, particularly the occurrence of full complement shifts (shifts that fulfilled their planned quota of RNs) and shifts with at least one agency RN staff or more. Of the 8841 shifts analysed, 3449 (39%) had full complement (Understaffing = 0). 6285 shifts (71%) had no agency staff (Agency ratio = 0), whilst 2556 shifts (29%) had zero values for both predictors (see Table 2). Out of the full complement shifts that have agency RN’s, the most prevalent staffing scenarios were those with a proportion of agency (0.1–0.2, (*N* = 193, 0.3–0.4, *N* = 150, 0.4–0.5 *N* = 196). In the low-understaffing category (25% or less), the most prevalent staffing scenarios were those with a 0.1–0.2 and 0.2–0.3 proportion of agency staff. In the high-understaffing category (25% or more), the most prevalent staffing scenarios were those with a 0.3–0.4 (*N* = 306) and 0.4–0.5 (*N* = 202) proportion of agency staff (see Table 2).

**Table 2:** Number of Shifts by Category of Agency and Understaffing Ratio All Settings

Proportion of Agency Staff	Understaffing Category			
	0 (Full complement)	0.01–0.24	≥25	Total
0	2556 (28.9%)	1519 (17.2)	2210 [17]	6285(71.1)
0.001–0.1	56(0.6)	143(1.6)	10(0.1)	209(2.36)
(0.1–0.2)	**193(2.2)**	**267 (3)**	107(1.2)	567(6.4)
(0.2,0.3)	**150(1.7)**	**221(2.5)**	99(1.1)	470(5.3)
(0.3,0.4)	**220(2.5)**	87(1)	**306(3.5)**	613(7)
(0.4,0.5)	**196(2.2)**	103(1.2)	**202(2.3)**	501(5.7)
(0.5,0.6)	12(0.1)	18(0.2)	9(0.1)	39(0.4)
(0,6, 0.7)	35(0.4)	9 (0.1)	82(0.9)	126(1.4)
(0.7,1)	9(0.1)	12 (0.1)	10(0.1)	31(0.3)

**Fig. 1:**
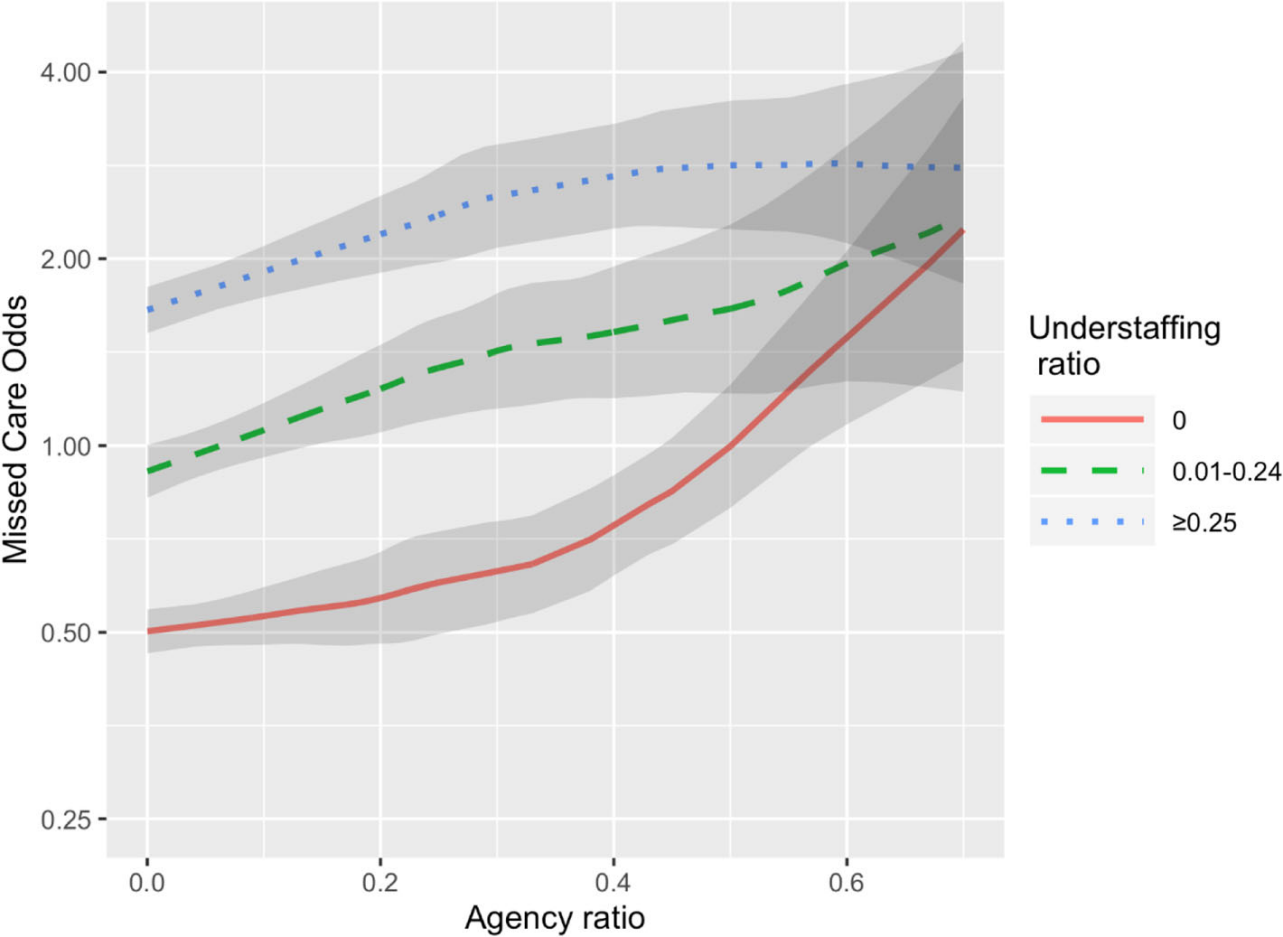
Estimated odds of any missed care event, by Agency ratio for three understaffing categories

In addition, we were interested in the relationship between the occurrence of any ‘care left undone’ events during the shift (a binary outcome) and two predictor variables, namely “Understaffing” (1 minus the ratio of actual to planned number of registered nurses) and “Agency ratio” (ratio of agency to total actual registered nurses working the shift). Table 3 presents the estimates and 95% confidence intervals for the probability of a ‘care left undone’ event at a range of values for Agency ratio in three grouped categories of understaffing ratio: 0, 0.01–0.24, and > =0.25. The data was categorised based on our interest in shifts that had no understaffing (understaffing = 0), low level of understaffing that happens due to unforeseen circumstances like for instance sick leave. Understaffing of 25% or more was considered moderate to high level of understaffing that might have significant impact on patient care. These categories each account for roughly a third of the shifts (*N* = 3427, 2379 and 3035 respectively). The odds calculations are based on the smoothed regression shown in Fig. 1, which presents this relationship graphically.

**Table 3:** Estimated odds of any missed care event, by agency ratio for three Understaffing categories

Odds of Care Left Undone, (95% CI)			
	Understaffing category		
Agency Ratio	0	0.01–0.24	≥0.25
0	0.50 (0.46, 0.54)	0.91 (0.82, 1.00)	1.65 (1.52, 1.80)
0.1	0.53 (0.48, 0.59)	1.06 (0.96, 1.17)	1.91 (1.73, 2.10)
0.2	0.57 (0.48, 0.67)	1.24 (1.05, 1.45)	2.19 (1.90, 2.53)
0.3	0.63 (0.52, 0.75)	1.42 (1.15, 1.76)	2.52 (2.08, 3.06)
0.4	0.74 (0.62, 0.90)	1.52 (1.19, 1.94)	2.72 (2.24, 3.30)
0.5	1.00 (0.79, 1.25)	1.66 (1.21, 2.27)	2.83 (2.23, 3.59)
0.6	1.49 (1.08, 2.06)	1.97 (1.27, 3.04)	2.85 (2.12, 3.83)
0.7	2.23 (1.37, 3.64)	2.34 (1.22, 4.48)	2.81 (1.82, 4.32)

The trend of odds of ‘care left undone’ in Fig. 1 shows that the odds of care left undone increases, with varying amounts, on shifts where there is increasing reliance on temporary staff, as indicated by increasing proportions of agency staff.

Our findings demonstrate that on full complement shifts with greater proportions of agency staff, the odds of care left undone increase. For instance, when comparing shifts that have no agency staff (Odds = 0.50, 95% CI, 0.46–0.54) to when the proportion of agency staff is 20% of the staffing (Odds = 0.57, 95% CI, 0.48–0.67), we see that the odds of missed care increase by 14% (OR = [1.14], 95% CI, 1.04–1.24). This difference becomes statistically significant on full complement shifts with 40% or more agency staff OR = [1.48], 95% CI, 1.40–1.70), (*p <* 0.05).

Similarly, where there is a 20% reliance on agency staff, and RN understaffing is 25% or less, the odds of ‘care left undone’ is 1.24 (95% CI, 1.05–1.45). The odds of ‘care left undone’ when agency usage is 20% rises where the RN staff ratio is less than 75% of planned full complement to 2.19 (1.9–2.53), *p* < 0.05.

Furthermore, considering a level of 25% or more understaffing, the odds of missed care events increase further still but only slightly with an increase in proportion of agency staff. Relating these results back to Fig. 1, whilst it can be observed that, within the range of 0–24% of understaffing, there is a steady increase in the occurrence of ‘care left undone’, relatively little further increase is observed in the 25% or more range. A simple linear logistic regression model confirms that the increase in missed care probability is statistically significant (*p* < 0.001) for both predictors separately, but the addition of significant quadratic and interaction terms confirms that the simple model does not adequately describe the patterns in the data.

## Discussion

Our study shows that both understaffing, and usage of agency nursing staff is prevalent, though not evenly distributed, across all adult acute care settings in the UK. Furthermore, our findings show that ‘nursing care left undone’ is also prevalent but not evenly distributed; in all adult acute settings, and that the odds increase with an increase in proportion of agency nursing staff. A similar trend, which is that higher levels of agency staff are associated with an increase in odds of care left undone, is most apparent on shifts with *no understaffing* (understaffing = 0) or *low level of understaffing* (less than 25%). In shifts with understaffing of 25% or more, the odds of ‘care left undone’ does not increase with higher proportions of agency staffing. This suggests that when RN staffing levels are considerably low agency nurses may ameliorate (but not reverse) the propensity for more care to be left undone.

The evidence presented in this study on the association between agency nurse staffing levels and risk of ‘care left undone’ has not been explored to this level of detail in previous research in the UK though some work has been undertaken in Emergency Care settings. A qualitative study of two emergency departments in a London-based hospital explored the impact of temporary staff on permanent clinical and management staff [18]. This study reported how staff and managers experienced significant stress when working with temporary staff. It describes the additional workload entailed in supervising temporary staff and how this leaves less time for urgent work and direct patient engagement. Linked to this, managers highlighted how employing temporary staff to achieve safe staffing numbers did not necessarily mean that the quality of service improved. They conceded that staff who were familiar with the clinical setting were preferred due to the stress and risks associated with new and unknown staff. Emergency Care then seems to pose a problem. Previous UK research has shown that 18% of nursing posts in Emergency Departments lack permanent staff [19]. It is perhaps no surprise then that our study found that emergency departments had the highest proportion of missed care.

The association between temporary staffing and risk of ‘care left undone’ has, however, been explored to some extent in other countries. Estabrooks al., (2005) examined relationships between Canada’s nursing workforce and hospital mortality and found that higher hospital death rates were associated with higher temporary staffing, highlighting the adverse effect of lack of continuity of care [20]. Pham et al., (2011) found that teamwork and communication were particularly challenging on shifts with high levels of temporary staff. In addition, the study examined the service quality and temporary staffing levels and showed that high workload and rising temporary staffing increased medication errors [21].

Similarly, we found that shifts with a fully planned complement of registered nurses but with a high proportion of temporary, agency nursing staff increased the odds of self-reported ‘care left undone’. These findings suggest that meeting planned staffing levels with agency RN staff is not as efficient as having a full complement of permanent staff when scheduling for safe and effective care. The importance of teamwork and communication in ensuring good quality care and avoiding ‘care left undone’ has been widely reported previously. Indeed Kalisch et al. identified relationships and communication as a significant antecedent to the process of missed care. A most recent study of agency nurses in critical care unit setting found self-reported lack of competence, self-efficacy and feelings of exclusion, which could compromise patient care [22]. Kalisch et al., (2011) have further utilised the MISSEDCARE tool in assessing variation across 10 US hospitals, finding that poor communication accounted for up to 80% of missed care. A review of teamwork studies demonstrated that collective orientation, shared mental models and closed loop communications were all necessary conditions for effective teamwork – all difficult to achieve with agency RN staff who may only be present for a single day or work episode [23]. The 2001 Audit Commission report on the use of temporary staff summarises this well when it concludes that even the most qualified temporary member of staff would struggle to perform due to unfamiliarity with the setting and their dependency on permanent staff for guidance [24].

It is therefore possible to speculate here that the presence of temporary agency staff contributes significantly to poor continuity of relationships and concomitant communication problems across nursing teams, culminating in an increased risk of ‘care left undone’ [10]. Although more research is required to examine the wider implications of temporary nurse staffing, findings here suggest that an emphasis on recruiting and retaining permanent registered nurses will likely be a better solution than short term, reactive responses to staff shortages.

### Limitations

There are several limitations to this study. In this analysis, we have only examined the association between staffing levels, proportion of temporary staff and self-reported nursing ‘care left undone’. Due to the nature of the recall, we have been unable to control for other variables that may have an impact on the risk of care left undone. From previous studies, we know that workload measured as hours per patient day (HPPD), size of setting, skill mix, time of shift, and unexpected increase in workload (either in terms of increased volume or increased level of patient acuity) have a significant relationship with risk of care left undone.

Furthermore, data accuracy is dependent on respondents’ ability to recall the most recent shift or work episode accurately. For this reason, there may be under and/or over-reporting of the ‘care left undone’ event, which we are unable to verify. In addition to this, we recognise that there are other factors, which may result in ‘care left undone’ which have not been incorporated into the statistical modelling here.

In addition, a significant number of respondents chose not to say if they had observed ‘care left undone’ or not. As such, we do not know if these data represent an under-reporting of the problem. Finally, the sample here was not a random or stratified sample but rather a convenience sample of those who chose to respond to a generic invitation. Despite the large number of participants, we cannot be sure that the sample is representative of the acute care sector nursing-workforce in terms of geographical distribution, personal characteristics (gender, ethnicity etc), and years of post-qualification experience and this has implications for claims that can be made about generalisability.

Lastly, the data within the survey was not linked to specific hospitals or Trusts for reasons of anonymity. For this reason, we could not conduct cluster-adjusted estimates analysis. However, the large final sample of 8841 were geographically dispersed across the four UK countries and it seems reasonable to expect that they were also well dispersed across organisations given the sampling strategy.

## Conclusion

These findings suggest a worrying prevalence of understaffing and care left undone in UK acute care sector nursing. They further demonstrate a relationship between both understaffing and high levels of agency nursing with increased care left undone and suggest that self-reported and self-perceived delivery of quality of care is jeopardised on wards with high levels of temporary staff due to care being left undone [17]. Given that care left undone has been associated with a variety of poor patient outcomes, including increased mortality [25], recent increases in the use of agency nurses in the UK (Addicott R, Maguire D, Jabbal J., 2015) are particularly concerning in terms of the ability for health organisations to deliver safe nursing care.

The work has relevance in terms of the allocation of health resources in workforce planning. Investment in policy and local management approaches that can improve RN recruitment, retention and reduce staff turnover are highly likely to improve the possibility of meeting the planned number of RN’s on a shift (through reducing sickness, reducing vacancies and increasing satisfaction) and concomitantly reducing the requirement for agency nursing staff. In turn, this would reduce the extent of care left undone and thereby enhance the effectiveness and safety of care and, ultimately, improve patient outcomes.

Future research should explore additional, setting-specific contributing factors to care left undone, such as variations in workload as well as specific aspects of and reasons for care left undone.

## Data Availability

The data that support the findings of this study are available from Royal College of Nursing but restrictions apply to the availability of these data, which were used after obtaining a Data Sharing Agreement and Ethical Approval for the current study, and so are not publicly available.

## Abbreviations

NHS: National Health Service
HPPD: Hours per patient per day
RN: Registered Nurse
RCN: Royal College of Nursing
ED: Emergency Department
IQR: Interquartile range
SD: Standard deviation
Mdn: Median
